# Salivary analysis to unveil the paradigma of stress of domestic horses reared in the wild

**DOI:** 10.1038/s41598-024-62172-2

**Published:** 2024-05-17

**Authors:** M. Bazzano, A. Marchegiani, F. La Gualana, B. Petriti, A. Spaterna, F. Laus

**Affiliations:** 1 School of Biosciences and Veterinary Medicine, University of Camerino, 62024 Matelica, MC Italy; 2 Department of Translational and Precision Medicine, Sapienza, University of Rome, Rome, RM Italy

**Keywords:** Stress, Horse, Welfare, Saliva, Cortisol, Alpha amylase, Butyrylcholinesterase, Biomarkers, Medical research

## Abstract

Horse welfare is the product of multiple factors, including behavioral and physiological adjustments to cope with stressful situation regarding environment and housing condition. Collectively, it is supposed that a horse kept in the wild has a lower level of stress than other housing system, and the aim of the present study was to investigate the level of stress in domestic horses reared in the wild and then moved to human controlled housing, through saliva analysis. Twelve clinically healthy Catria (Italian local breed) mares, usually reared in the wild, were moved into collective paddocks for a folkloric event. Saliva samples were obtained before and after the change of housing condition to evaluate stress biomarkers including salivary cortisol, salivary alpha-amylase, and butyrylcholinesterase (BChol). The mares were also scored using the Welfare Aggregation and Guidance (WAG) Tool to highlight the presence of abnormal behaviors. Despite the absence of differences in behavioral scores between wild and paddocks, salivary cortisol and BChol were found to be higher in the wild and lower when mares were moved to paddocks. The highest concentrations in stress biomarkers like salivary cortisol and BChol in the wild was unexpected, but the need for managing hierarchical relationships, and the exposure to feral animals, predators, and weather changes, might explain these findings. The overall results of the present study may provide further knowledge toward stress response in domesticated horses living in the wild moved to human controlled housing system.

## Introduction

In the last few years, an increasing interest has been pointed towards the use of saliva as a diagnostic fluid both in human and veterinary medicine^1–4^. It is well known that saliva composition can be affected by organic or psychological disorders and may reflect general metabolic changes^5^. In comparison with other biological samples (e.g. blood), saliva has the advantage of being easily collected by non-invasive and non-stressful procedures, which is extremely important when sampling animals^6^. Recently, some salivary markers, including cortisol and salivary alpha-amylase (sAA), have demonstrated the potential to make diagnosis of chronic stress or to discriminate between stress, anxiety, and depressive disorders in people^5,7^. Salivary cortisol has been used in most studies for the assessment of adrenocortical response to potentially stressful situations in horses^8^ so that it is recognized as an indicator of acute stress^9,10^. The concentration of alpha-amylase in saliva increases abruptly following both acute and chronic stress, which makes it an important salivary biomarker of stress^5^. Van Veen et al. (2008), suggested that sAA could be a potential salivary stress marker in people sensitive to a negative social assessment. Together with cortisol and sAA, salivary butyrylcholinesterase (BChol) is considered a reliable indicator of stress in equine^1,8,9,11^ and swine^12,13^.

Equids are prey animals, and they are inclined to live in group for safety. In order to maintain group membership and social arrangement, horses maintain their social relationship with the herd by scratching and rubbing, playing, resting, and following each other^14^. Their prey behavior influences also feed tendency, as for other species in nature; in fact, free roaming equines spend 16–20 h a day^15^ grazing large amounts of high fiber food^16^. It has been reported that horses continuously living on pasture with conspecifics display a more natural expression of the species’ behaviors such as feeding and social interactions^17,18^ and less health impairments^19^, suggesting a better welfare state than horses kept in individual boxes^20,21^.

Chronic stress induced by missing contact with other horses or by diseases causing pain (e.g. chronic lameness) have the potential of affecting the horse’s physical health. In this perspective, the evaluation of stress biomarkers can be relevant when assessing horse welfare, health, or disease predisposition. According to some Authors, domestic horses are informative models to investigate the impact of stress on the HPA (hypothalamic–pituitary–adrenal) axis, as they commonly experience conditions impairing their welfare related to social, spatial, and feeding restrictions^22^. HPA axis response to acute stress has been studied by several authors but its interpretation in cases of prolonged stress is far from straight forward^23–26^. It is assumed that stabled horses are more prone to be stressed and to develop stereotypic and redirected behaviors that are generally used as an indicator of poor welfare associated with stabling^27^. Stress and abnormal behaviors are less likely to develop in animal that spent significantly longer time outside or that are kept in pasture^28^.

Therefore, stress in wildlife can be considered different from than in related domestic species and free-ranging animals experience differently stress from captive ones^29^. In this article, we have attempted to depict the difference in stress response in free-ranging domesticated horses (usually living in the wild) and then exposed to a potential stressful condition like a local event, by determining changes in salivary cortisol, sAA, and BChol.

## Results

According to the WAG tool, all the mares expressed the same behavioral score, resulting in grade A (good welfare) both in the wild and in paddock, irrespective of the different setting. The mouthpiece used for sampling was well tolerated by all horses (no signs of discomfort or stress were displayed during collection) and allowed an easy-going collection in all horses.

**Figure 1:**
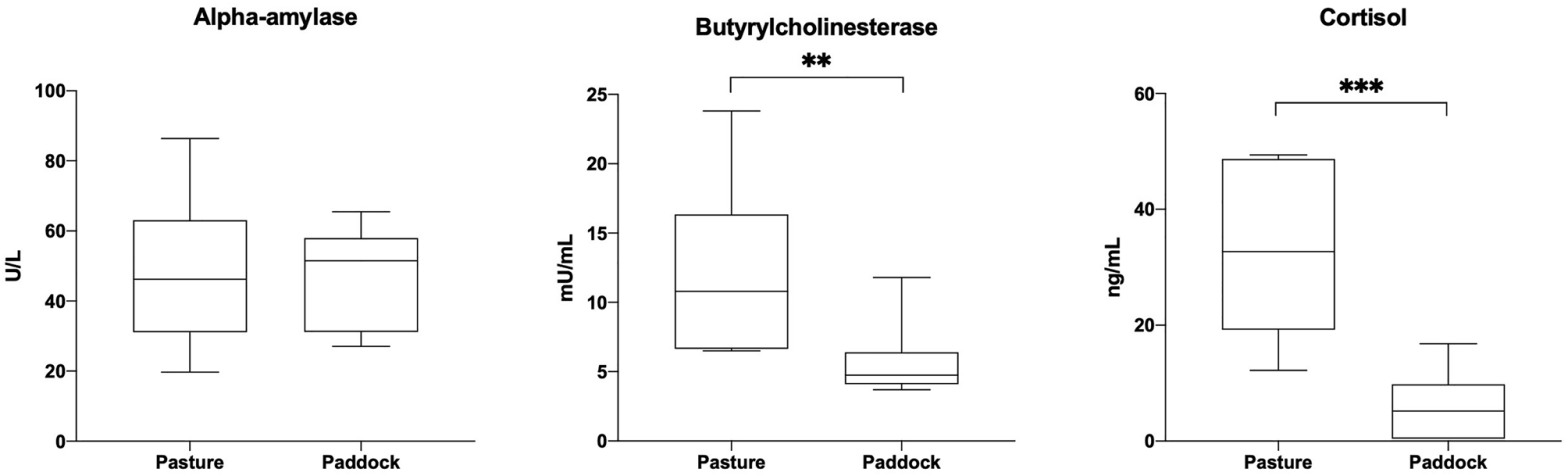
Salivary alpha-amylase (sAA) activity, butyrylcholinesterase (BChol), and cortisol from the same horses stabled in pasture and paddock. Asterisk indicates statistically significant differences (**p < 0.01, ***p < 0.001) between groups.

The concentrations of sAA, BChol, and cortisol in saliva samples collected from the same horses in the wild and in paddock are reported in Table 1. The concentration of sAA were similar between pasture and paddock (46.25 ± 19.44 and 47.52 ± 13.11 U/L, respectively; *p* = 0.7742), with a wider range (from 19.70 to 86.40 U/L) in the wild compared to paddock housing (from 27.10 to 65.50 U/L) (Fig. 1). BChol was found to be higher during pasture stable and lower in a statistically significant way when horses moved to paddock (12.44 ± 6.30 and 5.58 ± 2.39 mU/mL, respectively; *p* = 0.0068). The same difference was registered for cortisol, for which significant higher salivary concentration was found when horses were stabled in pasture in comparison with paddock (33.59 ± 13.44 and 5.99 ± 5.70 ng/mL, respectively; *p* = 0.0002).

**Table 1:** Descriptive statistics for salivary biomarkers.

	Wild				Paddock			
	Mean ± SD	Min	Max	Range	Mean ± SD	Min	Max	Range
sAA	46.25 ± 19.44	19.70	86.40	66.70	47.52 ± 13.11	27.10	65.50	38.40
BChol **	12.44 ± 6.30	6.50	23.80	17.30	5.58 ± 2.39	3.70	11.80	8.10
Cortisol ***	33.59 ± 13.44	12.20	49.40	37.20	5.99 ± 5.70	0.40	16.80	16.40

Data are expressed in U/L, mU/mL, and ng/mL for salivary alpha-amylase (sAA), butyrylcholinesterase (BChol), and cortisol, respectively. Asterisk indicates statistically significant differences (**p < 0.01, ***p < 0.001) between groups.

*min* minimum concentration, *max* maximum concentration.

## Discussion

In the planning phase of the study, we assumed that the modification of horse environment from wild setting to paddock restraining could have had a direct influence on both horse behavior and stress parameters. Unexpectedly, the mares did not change any behavioral aspect when observed in collective paddocks during the event compared to wild environment.

A different result was obtained recently by Ruet and collaborators^21^ that studied the effect of offering horses a short-term period on pasture with conspecifics to alleviate the impact of long-term deprivation triggered by box shelter. When on pasture, horses increased the expression of natural behaviors which may have not been easily expressed when stabled in boxes without displaying any stereotypies and aggressive behaviors towards humans. Authors concluded that pasture can positively influence the welfare state of horses usually stabled individually, but also that several days of adaptation are needed, probably due to the novelty of the environmental and the needing to establish social conditions^21^.

Conversely from Ruet and collaborators, in the present study the behavioral evaluation of horses did not change when passing form wild to paddock. A possible explanation to our finding could be that the mares were moved into collective paddocks and not in single boxes, maintaining their herd; in addition, it is not possible to exclude a possible effect of different data collection and behavior analysis methods between the two studies.

Differently from behavioral observations, objective salivary parameters of horse stress namely BChol and Cortisol significantly changed during the study, while sAA remained unchanged. Regarding sAA, it is considered a potential salivary stress marker of acute and chronic stress in people, especially in those sensitive to a negative social assessment^30,31^. In horses experimenting an acute stress, for example immediately after a sudden scare, sAA increase, to return at lower level after few minutes^32^. Since in our study horses were not exposed to an acute stress, the lack of change we observed could be due to the lack of reliability of sAA in evaluating non-acute stress in horses. BChol, a serine hydrolase biochemically related to the cholinergic enzyme acetylcholinesterase, is widely distributed in the nervous system, and it seems to be involved in neural function serving as a co-regulator of cholinergic transmission. The function of BChol in saliva is not fully understood but it has been associated with acute stress in humans, rats, and pigs^33^. In our study, Bchol salivary concentrations were significantly higher in saliva samples collected in the wild compared to samples collected on the same mares restrained in paddocks. A similar difference was observed in salivary cortisol showing the highest mean values when the mares stayed in the wild. Salivary cortisol represents the unbound biologically active fraction of total plasma cortisol, that is mainly bound to carrier proteins, thus reflecting the hypothalamic–pituitary–adrenal (HPA) axis acute activation^34,35^.

Apart from medical situations that can cause a variation in its secretion (i.e. equine Cushing’s disease, inflammatory and infectious diseases, as well as metabolic imbalances and dehydration), cortisol levels may fluctuate in different conditions such as changes in location, handling procedures, training, and racing activities^36^.

Blood and salivary cortisol are suitable tools for evaluating acute stress response, while are not reliable markers of chronic stress due to intrinsic physiological secretion features^37^. In fact, salivary cortisol is generally used for the detection of acute stress while hair cortisol concentration offers an overview of chronic stress, with no impact of short-term oscillations due to acute stressors. As a limitation of the present study, hair cortisol concentration has not been evaluated and it would have been beneficial in the detection of different stress response in horses. In addition, it is not possible to exclude the presence of other factors apart from environment that may have caused an increase in cortisol levels.

A possible explanation of the findings of the present study could be an adaptive approach called “coping strategy” that defines the behavioral and physiological struggles of animal to face a new, potentially stressful situation. The coping strategy attracts the interest of different authors dealing with applied horse welfare and behavior^38^. When a change in environment occurs, as in the present study, animals need to cope with their environment using both behavioral and physiological stress responses. Different parameters are related to such coping strategy and may be monitored over time such as changes in form and frequency of behavioral response, changes in animals-based index (such as heart rate, body temperature, hormones, neutrophils and lymphocytes ratio, and other blood indicators). In this context, the discovery of new biomarkers of stress represents a crucial milestone for understanding the individual adaptive capacity to stressful events in domesticated and free-ranging animals^39^.

Environment has a major impact on horse welfare and behavior (influencing for example the chance of feeding, protection from weather condition, and sleep-awake cycle)^40^ and horse may reveal specific behavior patterns to adapt to challenging situations especially in man-made environment^41^.

The results of the present study provide new knowledge in the multidimensional approach toward horse behavior understanding, hopefully providing valuable information for specific, possibly challenging situations. Animals living in man-made environments (as paddocks in the present study), are believed to be influenced by different kinds of stressor agents that causes adaptive responses^42^. On the other hand, it could also be hypothesized that although life in the wild allows horses to shows their natural behavior in freedom, at the same time this could represents a stress factor, linked to the need for managing hierarchical relationships within the herd, and the exposure to feral animals, predators, and weather changes, might explain these findings.

We aimed to appraise the relationship between behavioral and physiological factors involved in the horse stress response and, surprisingly, salivary biomarkers revealed that stress level was higher in the wild.

It should also be underlined that a large heterogeneity between the studies exists, because of the lack of standardized methods for measuring salivary cortisol, heterogeneity of studied populations and the bias of risk factors. In human being, for example, adolescents with severe symptoms of depression have not different concentration of salivary cortisol when compared to adolescents with mild symptoms of depression but had a significantly higher ratio of morning to evening levels of salivary cortisol^43^. This should be taken in account in future studies also in veterinary research. The findings of the present study are related to a typical Italian horse breed and the same evaluation considering different breeds/attitudes is warmly welcome, to provide more details to interpret horse behavior. Exploring coping strategies in horses and in any captive animal can be successful when a multidimensional approach including behavioral, neural, hormonal, and hematological measures is considered. Knowledge on stress and related coping styles can provide valuable information to prevent potentially challenging situations, in order to maximize animal welfare.

## Materials and methods

### Animals and behavioral assessments

**Figure 2:**
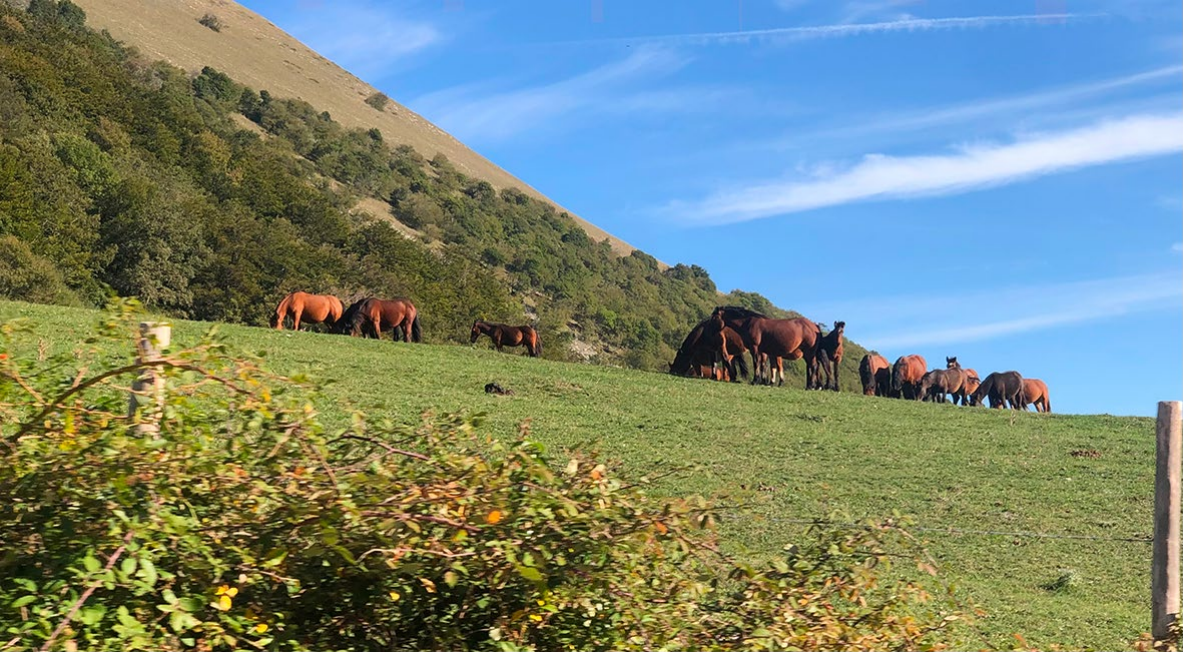
Catria horses.

A total of 12 clinically healthy mares were included in the study with informed owners’ consent. The animals belonged to the Italian local breed Catria Horse known for its endurance, resistance, and well-balanced attitude (Fig. 2). Horses’ mean age was 6.5 ± 6 years, and BCS ranged from 6 to 7 out of 9 points scale^44^. As inclusion criteria, all animals were domesticated and accustomed to handling before the beginning of the study, and they were maintained at pasture in the wild throughout the year. The study design consisted of behavioral evaluations and saliva collections on the same animals in the wild (usual environment) and in collective paddocks on occasion of a two-day folkloric event (Fiera del cavallo del Catria, which is held in central Italy in October). Samplings were conducted during the same week: one day prior horses were moved from pasture to the fair and then three days after, in order to allow them to familiarize with the paddock housing.

Behavioral indicators were scored using the Welfare Aggregation and Guidance (WAG) Tool^45^ evaluating the presence of abnormal behaviors like aggressiveness, unresponsiveness to the environment, hypervigilance, etc. All assessments were performed by an experienced observer.

The first saliva sampling was performed with the free-ranging mares in their usual habitat (wild). The second sampling was collected from the same mares moved in paddocks on occasion of a local event. When in paddock, horses were fed with ad libitum polyphyte hay. To avoid any interference on studied parameters, the procedures of the second time point were performed after 48 h from relocation to allow the animals to familiarize with the new condition.

**Figure 3:**
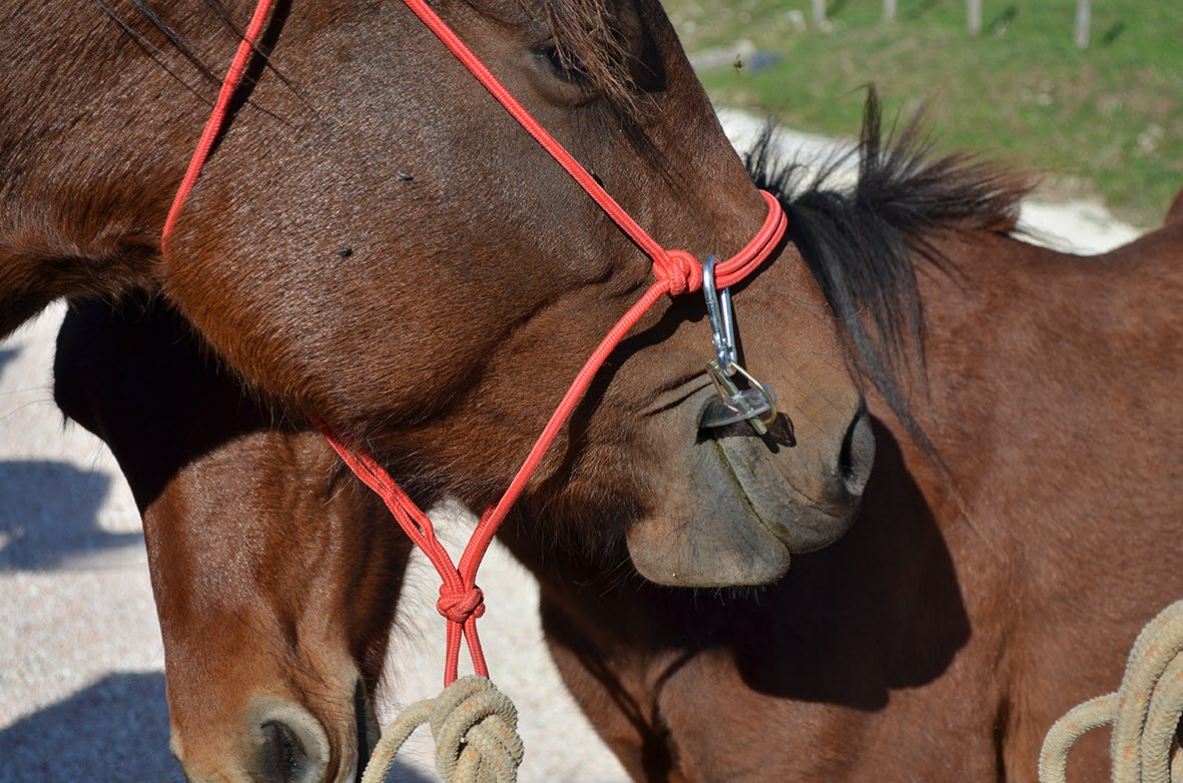
Mouthpiece during saliva collection.

Saliva samples were collected in the morning (10:00 a.m.–11:00 a.m.), using cotton swabs (Salivette^®^ Sarstedt AG & Co., Nümbrecht, Germany) inserted into a customized hollow mouthpiece that horses chewed for 5 min (Fig. 3). After each collection, cotton swabs were removed from the mouthpiece by means of surgical clamps, cooled immediately and delivered to the laboratory within 2 h. After behavioral evaluations and saliva collections, the mares were examined by an experienced clinician to verify the health conditions of each subject.

All experimental procedures were approved by the Institutional Animal Care and Use Committee of Camerino University (Registration number: 10/2023) and were in accordance with the standards recommended by the EU Directive 2010/63/EU for experiments on animals. The experiment complied with ARRIVE guidelines.

### Laboratory analysis

Saliva samples were obtained by centrifugation at 3000 g for 20 min (Universal 32, Hettich Zentrifugen, Tuttlingen, Germany) of Salivette^®^ tubes (SARSTEDT AG & Co. KG, Nümbrecht, Germany) and 0.5 mL aliquots of saliva were stored at − 20 °C until analysis. At the day of the analysis saliva samples were thawed and centrifuged (10 min at 1000 g) to eliminate debris residues. Surnatants were collected and cortisol, BChol, and amylase levels were measured by using Horse cortisol ELISA Kit, Horse Cholinesterase ELISA Kit, Horse Amylase ELISA kit (Bioassay Technology Laboratory), respectively, following the Manufacturer’s instructions. The absorbance was determined with Thermo Scientific Multiskan Sky amounted to 450 nm.

### Statistical analysis

Statistical analysis was performed using GraphPad Prism version 8.2.1 for macOS, GraphPad Software, La Jolla California USA. sAA activity, salivary BChol and cortisol concentrations were checked for normality using Shapiro–Wilk test, showing sAA and cortisol normally distributed while BChol was not. Then, paired t-test was applied to alpha-amylase and cortisol, and Wilcoxon test to BChol to assess if concentrations of salivary biomarkers were statistically different between pasture and paddock. Values of *p* < 0.05 were considered significant.

A post hoc analysis (G*Power 3.1.9.6 for Mac OS) was performed to assess the significance level and power obtained. Including a number of twelve horses gave a significance level of α = 5% (*p* < 0.05) and a power of 80%.

## Data Availability

Data generated during the study are available upon author’s request (please email to andrea.marchegiani@unicam.it).
